# Association of TNFAIP8 gene polymorphisms with endometrial cancer in northern Chinese women

**DOI:** 10.1186/s12935-019-0827-9

**Published:** 2019-04-23

**Authors:** Tianbo Liu, Liangliang Jiang, Libo Yu, Tingting Ge, Jing Wang, Hongyu Gao

**Affiliations:** 1 Department of Gynecology, Harbin Medical University Cancer Hospital, Harbin Medical University, 150 Haping Road, Harbin, 150081 China; 2 Department of Gastroenterologic Surgery, Harbin Medical University Cancer Hospital, Harbin Medical University, 150 Haping Road, Harbin, 150081 China

**Keywords:** TNFAIP8, Polymorphism, Endometrial cancer, Protein expression, Susceptibility

## Abstract

**Background:**

Tumor necrosis factor-a-induced protein 8 (TNFAIP8) presented a elevated expression in endometrial cancer (EC). However, the relationship of TNFAIP8 gene polymorphisms with EC risk remains unclear. This case–control study aimed to investigate the effect of single nucleotide polymorphisms (SNPs) in TNFAIP8 on northern Chinese women with EC.

**Methods:**

SNP rs11064, rs1045241, and rs1045242 in TNFAIP8 were successfully genotyped in 248 cancer-free controls and 226 ECs by SNaPshot method, respectively. Logistic regression was performed to assess relationship of SNPs with EC risk. The relationships of SNPs with clinicopathological variables were evaluated by Chi-square test or Student’s t-test or Fisher’s text.

**Results:**

The minor alleles of rs11064 in TNFAIP8 were strongly associated with EC risk, with adjust odds ratio (OR) of 1.719 (95% CI 1.180–2.506, P = 0.005). The minor allele of rs1045242 in the TNFAIP8 gene was strongly associated with with EC risk (adjust OR: 1.636, 95% CI 1.107–2.417, P = 0.014). rs11064 SNPs correlated with TNFAIP8 protein expression in EC (P = 0.015). For rs1045242, patients with AG + GG presented higher TNFAIP8 protein expression than that with AA (P = 0.020). It also showed that SNP rs11064 was associated with advanced FIGO stage (P = 0.001), deep myometrial invasion (P = 0.047), and lymph node metastasis (P = 0.048) under the codominant model in ECs.

**Conclusions:**

SNP rs11064 in TNFAIP8 increased EC risk and significantly related with its protein expression in northern Chinese women.

**Electronic supplementary material:**

The online version of this article (10.1186/s12935-019-0827-9) contains supplementary material, which is available to authorized users.

## Background

Endometrial cancer (EC) presents the fourth most often cancer among female around the world [1]. Presently, the incidence of EC continues to rise in developing countries, and the age of diagnosis is getting younger. Besides, all kinds of genetic mutations and abnormal activation of relevant signaling pathways are intimately associated with the occurrence and development of EC [2]. Therefore, it is needful to investigate the underlying mechanisms of these genes as clinical molecular markers in EC.

TNFAIP8 was first found in primary human head and neck squamous cell carcinoma (HNSCC) cell line and its matched metastatic cell line which were from the same patient through analysis of the expression profile [3]. Accumulating data indicates that upregulation of TNFAIP8 participated in tumor cell progression, proliferation, invasion, migration, apoptosis, and chemotherapy resistance in different types of tumor [4–18]. Previously, we have demonstrated elevated expression of TNFAIP8 mRNA and protein in tissues with EC, and its upregulation negatively affect prognosis of EC [10]. Nevertheless, the potential molecular mechanism of the abnormality of TNFAIP8 in EC remains to be unclear. As is known to all that the polymorphisms of gene could influence gene expression. Therefore, we assume that the polymorphisms of TNFAIP8 may be correlated with protein expression and may impact EC risk and prognostic factors.

We aimed to explore TNFAIP8 polymorphisms and their association with EC risk. We also examined the association between TNFAIP8 polymorphisms and prognostic factors in EC.

## Materials and methods

### Study population

This case–control study included 226 cases with EC and 248 cancer-free control samples. All subjects were ethnically homogenous Chinese and resided in the Heilongjiang province of China. Cases primarily diagnosed with EC were treated with hysterectomy, bilateral salpingooophorectomy, pelvic and/or paraaortic lymphadenectomy, partial omentectomy and peritoneal washing for cytology at the Department of Gynecology, Harbin Medical University Cancer Hospital. All the patients did not have radiotherapy or chemotherapy history before surgical operations. The participants were genetically not related in three generations. After providing informed consent, each participant was interviewed to collect detailed information on demographic characteristics and provided 5 mL venous blood from September 2015 to February 2017. This study was approved by the Medical Ethics Committee of Harbin Medical University, Harbin, China.

### SNPs genotyping of TNFAIP8

We performed a combined analysis of functional significance and Tag SNP strategies to select three potential functional SNPs of the TNFAIP8 gene from the dbSNP and HapMap databases. The three SNPs were rs11064, rs1045241, and rs1045242, respectively. Genomic DNA was obtained from the whole blood, and was isolated from EDTA anti-coagulated whole blood using the AxyPrep Blood Genomic DNA Miniprep Kit (Axygen Biotechnology, Union City, CA, USA). The SNaPshot SNP assay was carried out to detect the dimorphism at the seven SNP loci. The resulting data were analyzed with GeneMapperTM 4.0 Software (Applied Biosystems, Foster City, CA, USA). To ensure quality-control, genotyping was done without knowledge of case/control status of the subjects, and a 5% random sample of cases and controls was genotyped twice by different persons; the reproducibility was 100%.

### Immunohistochemistry (IHC)

All 226 primary EC tissues were stained by IHC. IHC staining for TNFAIP8, estrogen receptor (ER), progesterone receptor (PR), P53 and Ki67 were performed using the Two-Step IHC Detection Reagent (PV-6001) kit (Zhong Shan Golden Bridge Biological Technology Inc., Beijing, China). The antibodies dilutions and sources were as follows: rabbit polyclonal antibodies for TNFAIP8 (1:100; Abcam), monoclonal antibodies for ER (1:100; Ventana), PR (1:70; Dako), P53 (1:600; Dako) and Ki67 (1:250; Dako). TNFAIP8 status was scored as our previous research [7]. ER and PR status were scored by the current American Society of Clinical Oncology (ASCO)/College of American Pathologists (CAP) guidelines [19]. All the samples were considered to be positive for ER or PR when at least 1% of the tumor cell nuclei were stained. The samples with nuclear staining in at least 10% of tumor cells were considered positive for P53 [20]. And the positivity threshold for Ki67 was more than 14% of tumor cells with stained nuclei [21].

### Statistical analysis

The genotype frequencies were tested for Hardy–Weinberg equilibrium using the Chi-square test among the controls. Differences between cases and controls in demographic characteristics were evaluated by the Chi-square test or Fisher’s text (for categorical variables) or Student’s t-test (for continuous variables). The association between TNFAIP8 gene polymorphisms and protein expression was evaluated by the Chi-square test or Fisher’s text. Associations between genotypes and EC risk were estimated by computing odds ratios (ORs) and 95% confidence intervals (CIs) from logistic regression with adjustment for age, smoking history, BMI, and menopausal status.

## Results

### Subject characteristics

The 226 cases and 248 controls were similar with regard to age at interview, parity, diabetes and smoking history (Table 1). However, there were significant differences between cases and controls in the BMI (P < 0.001), age at menarche (P = 0.003), menopausal status (P < 0.001), and hypertension (P < 0.001).

**Table 1:** Characteristics of 226 endometrial cancer cases and 248 cancer-free controls

Characteristics	Cases	Controls	*P* ^a^
Age	53.92 ± 8.396	53.44 ± 8.692	0.545
BMI	25.59 ± 3.576	23.79 ± 4.919	< 0.001
Age at menarche	14.60 ± 1.497	15.06 ± 1.886	0.003
Menopausal status			< 0.001
Pre-menopausal	86	195	
Post-menopausal	140	53	
Parity			0.905
Nulliparity	30	32	
Multiparity	196	216	
Hypertension			< 0.001
No	163	216	
Yes	63	32	
Diabetes			0.053
No	200	232	
Yes	26	16	
Smoking history			0.759
No	206	228	
Yes	20	20	

*BMI* body mass index

^a^Two-sided Chi-squared test or Fisher’s test or student’s t test

### Relationships of TNFAIP8 SNPs with EC risk

The allele and genotype distributions for all SNPs in cases and controls were shown in Table 2. The observed genotype frequencies of three SNPs followed Hardy–Weinberg equilibrium among the controls (P > 0.05 for all three SNPs).

**Table 2:** Genotype frequencies of TNFAIP8 gene polymorphism among patients and controls and their associations with the susceptibility of endometrial cancer

Variables	Cases (%) n = 226	Controls (%) n = 248	*P* ^a^	Crude OR (95% CI)	*P*	Adjust OR (95% CI)	*P* ^b^
Genotypes							
rs11064			0.029				
AA	138 (61.1)	178 (71.8)					
AG	70 (31.0)	60 (24.2)		1.505 (0.998–2.268)	0.051	1.571 (0.972–2.539)	0.065
GG	18 (7.9)	10 (4.0)		2.322 (1.039–5.190)	0.040	2.582 (1.034–6.445)	0.042
AG + GG	88 (38.9)	70 (28.2)		1.622 (1.104–2.382)	0.014	1.778 (1.134–2.782)	0.012
rs1045241			0.529				
CC	143 (63.3)	167 (67.3)					
CT	71 (31.4)	72 (29.1)		1.152 (0.775–1.712)	0.485	1.144 (0.906–2.299)	0.122
TT	12 (5.3)	9 (3.6)		1.557 (0.638–3.802)	0.331	1.980 (0.699–5.607)	0.198
CT + TT	83 (36.7)	81 (32.7)		1.197 (0.819–1.748)	0.353	1.501 (0.961–2.344)	0.074
rs1045242			0.197				
AA	144 (63.7)	177 (71.4)					
AG	74 (32.7)	65 (26.2)		1.399 (0.939–2.086)	0.099	1.568 (0.980–2.507)	0.061
GG	8 (3.6)	6 (2.4)		1.639 (0.556–4.831)	0.370	2.695 (0.799–9.084)	0.110
AG + GG	82 (36.3)	71 (28.6)		1.420 (0.965–2.089)	0.076	1.651 (1.049–2.599)	0.030
Alleles							
rs11064							
A	346 (76.5)	416 (83.9)					
G	106 (23.5)	80 (16.1)	0.005	1.593 (1.153–2.201)	0.005	1.719 (1.180–2.506)	0.005
rs1045241							
C	357 (79.0)	406 (81.9)					
T	95 (21.0)	90 (18.1)	0.265	1.200 (0.870–1.656)	0.265	1.457 (1.001–2.120)	0.050
rs1045242							
A	362 (80.1)	419 (84.5)					
G	90 (19.9)	77(15.5)	0.077	1.353 (0.968–1.892)	0.077	1.636 (1.107–2.417)	0.014

*BMI* body mass index, *OR* odds ratio, *CI* confidence interval

^a^Two-sided Chi-squared test

^b^Data were calculated by logistic regression, adjusted for age, smoking history, BIM, menopausal status

The results showed that the minor allele of rs11064 in the TNFAIP8 gene was strongly associated with EC in patients (crude OR: 1.593, 95% CI 1.153–2.201, P = 0.005 and adjust OR: 1.719, 95% CI 1.180–2.506, P = 0.005). There was no association between the minor allele of rs1045242 in the TNFAIP8 gene and EC risk (crude OR: 1.353, 95% CI 0.968–1.892, P = 0.077). After adjusting for age, BMI, menopausal status, and smoking history, however, we observed that the minor allele of rs1045242 in the TNFAIP8 gene related significantly with EC risk (adjust OR: 1.636, 95% CI 1.107–2.417, P = 0.014).

We further analyzed the effect of the genotypes of these SNPs under three different genetic models. In the logistic regression models, compared with AA genotype of rs11064, GG genotypes was associated with an increased risk of EC (adjusted OR = 2.582, 95% CI 1.034–6.445, P = 0.042). This SNP was also related with an increased risk of EC under a dominant model (GG + AG vs. AA, adjust OR = 1.778, 95% CI 1.134–2.782, P = 0.012). Compared with the rs1045242 AA genotype, the AG and GG genotype possibly conferred increased risk for EC in the dominant model (adjust OR = 1.651, 95% CI 1.049–2.599, P = 0.030). However, no significant association with EC risk was observed for rs1045241 in the TNFAIP8 gene.

### Stratified analysis by age, smoking history, BMI, and menopausal status

The results of stratified analyses are shown in Additional file 1: Table S1, Additional file 2: Table S2, Additional file 3: Table S3, and Additional file 4: Table S4. For the patients whose age was greater than 54, in the dominant model, combined genotypes (AG + GG) of rs11064 had a 1.899-fold increase EC risk compared with the genotype AA (OR = 1.899, 95% CI 1.051–3.430, P = 0.034). However, for patients whose age was no more than 54, we did not observe the association between genotypes and EC risk. For patients who have no smoking history, the minor allele of rs1045242 significantly increased EC risk under co-dominant and dominant models (P < 0.05). Furthermore, for patients whose BMI was greater than 25, in the dominant model, combined genotypes (AG + GG) of rs11064 had a 2.358-fold increase EC risk compared with the genotype AA (OR = 2.358, 95% CI 1.133–4.906, P = 0.022). Interestingly, when evaluating menopausal status, we found that the minor allele of rs1045242 strongly increased EC risk under co-dominant and dominant models (P < 0.05) in patients who were at pre-menopausal. In addition, we observed that for patients who were at pre-menopausal, combined genotypes (AG + GG) of rs11064 had a 1.747-fold increase EC risk compared with the genotype AA (OR = 1.747, 95% CI 1.027–2.972, P = 0.040).

### Associations between TNFAIP8 SNPs and TNFAIP8 protein expression

**Fig. 1:**
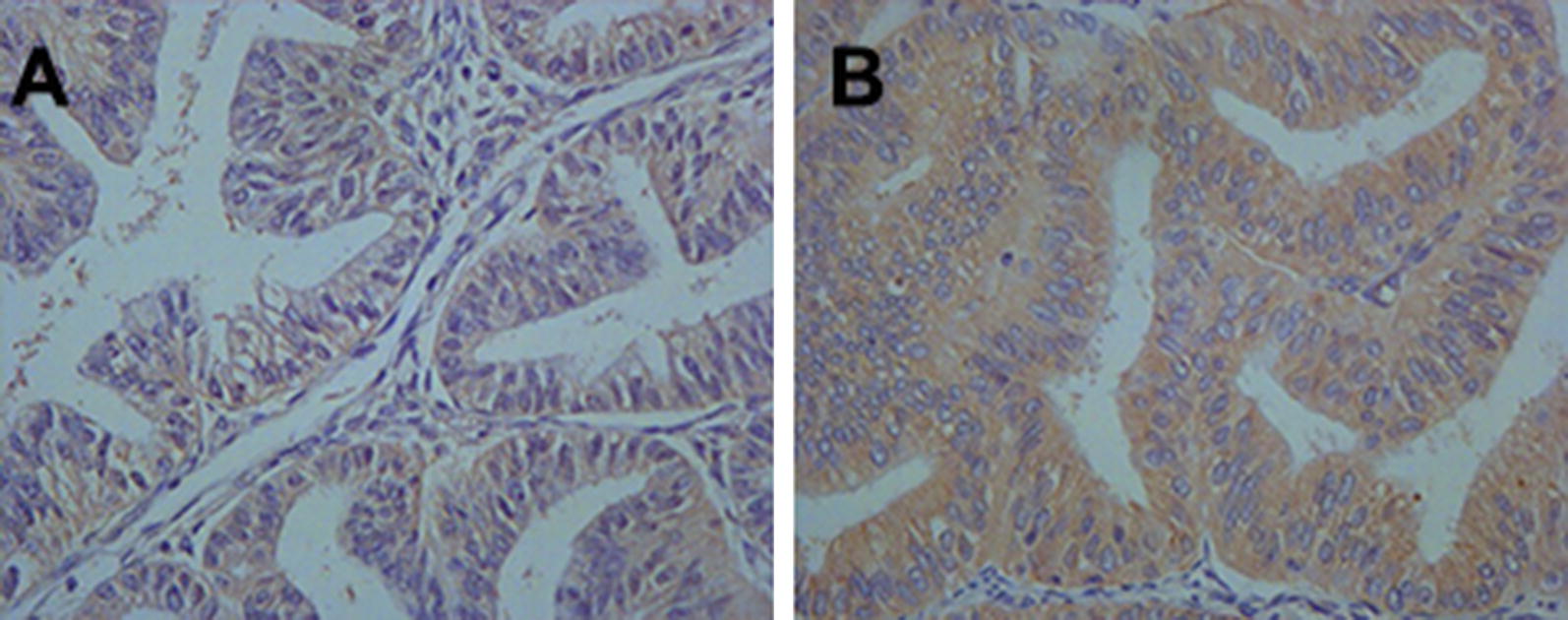
Immunohistochemical staining of TNFAIP8 protein in endometrial cancer tissues. TNFAIP8 immunoreactivity was observed mainly in the cytoplasm. Staining for each specimen is shown at magnification ×400. TNFAIP8 protein low expression slides (**a**); TNFAIP8 protein high expression slides (**b**)

The TNFAIP8 protein expression in EC tissue was shown in Fig. 1, and the immunostaining were localized within the cytoplasm of tumor cells. SNP rs11064 was significantly associated with TNFAIP8 protein expression under the codominant model (P = 0.005, Table 3). Moreover, patients with genotypes AG and GG were significantly associated with increased TNFAIP8 protein expression under the dominant model (P = 0.015, Table 2).

**Table 3:** Association of different SNPs in the TNFAIP8 gene with TNFAIP8 protein expression from 226 patients with endometrial cancer

SNP	TNFAIP8 expression		*P* ^a^
	High (%) (n = 85)	Low (%) (n = 141)	
Genotypes			
rs11064			
AA	42 (49.4)	96 (68.1)	
AG	33 (38.8)	37 (26.2)	
GG	10 (11.8)	8 (5.7)	0.015
AG + GG	43 (50.6)	45 (31.9)	0.005
rs1045241			
CC	48 (56.5)	95 (67.4)	
CT	32 (37.6)	39 (27.7)	
TT	5 (5.9)	7 (4.9)	0.229
CT + TT	37 (43.5)	46 (32.6)	0.099
rs1045242			
AA	46 (54.1)	98 (69.5)	
AG	34 (40.0)	40 (28.4)	
GG	5 (5.9)	3 (2.1)	0.060
AG + GG	39 (45.9)	43 (30.5)	0.020

^a^Two-sided Chi-squared test or Fisher’s test

SNP rs1045242 was correlated with TNFAIP8 protein expression under the dominant model (P = 0.020, Table 3). However, the correlation with TNFAIP8 protein expression was not found in the codominant model (P = 0.060, Table 3).

No significant association was observed between SNP rs1045241 and TNFAIP8 protein expression under the codominant model and the dominant model (P = 0.229; P = 0.099, respectively, Table 3).

### Associations between TNFAIP8 SNPs and the clinical characteristics of EC cancer

The association of rs11064, rs1045241 and rs1045242 polymorphisms with clinicopathological characteristics is shown in Table 4. SNP rs11064 had a significant association with FIGO stage (P = 0.011), depth of myometrial invasion (P = 0.047), and lymph node metastasis (P = 0.048) under the codominant model. However, these correlations were not found under dominant model. For SNP rs1045242, it was found that the patients with genotypes AG and GG were more likely to have advanced FIGO stage compared to the patients with genotype AA (P = 0.041). Furthermore, we observed the patients with genotypes AG and GG were more likely to have deeper myometrial invasion (P = 0.034). Yet, these correlations were not found under codominant model. No significant associations could be found between these two SNPs and EC patients’ histologic grade, histological type, LVSI, CA-125 level, P53, ER, PR, and Ki67 expression. Additionally, in this study, there were no significant associations between rs1045241 and all disease characters.

**Table 4:** The association between rs11064, rs1045241 and rs1045242 and clinicopathological characteristics of endometrial cancer

Characteristics	rs11064				*P* ^a^	rs1045241				*P* ^a^	rs1045242				*P* ^a^
	AA	AG	GG	AG + GG		CC	CT	TT	CC + CT		AA	AG	GG	AG + GG	
FIGO stage					0.011					0.174					0.103
I	108	60	10	70	0.425	115	54	9	63	0.075	114	58	6	64	0.041
II	23	6	3	9		22	8	2	10		24	7	1	8	
III–IV	7	4	5	9		6	9	1	10		6	9	1	10	
Histologic grade					0.718					0.678					0.658
G1	56	29	5	34	0.966	57	28	5	33	0.554	56	30	4	34	0.383
G2	60	29	11	40		66	28	6	34		68	29	3	32	
G3	22	12	2	14		20	15	1	16		20	15	1	16	
Histological type					0.445					0.469					0.464
EC	112	57	17	74	1.000	114	61	11	72	0.182	116	62	8	70	
Non-EC	26	13	1	14		29	10	1	11		28	12	0	12	
Depth of MI					0.047					0.153					0.088
< 50%	95	53	17	70	0.077	98	98	8	65	0.114	97	60	6	66	0.034
≥ 50%	43	17	1	18		45	45	4	18		47	14	2	16	
LVSI					0.708					0.338					0.562
No	118	63	16	79	0.350	122	122	10	75	0.274	123	67	7	74	0.297
Yes	20	7	2	9		21	21	2	8		21	7	1	8	
LN metastasis					0.048					0.702					0.934
No	122	62	12	74	0.351	123	123	10	73	0.679	124	65	7	72	0.718
Yes	16	8	6	14		20	20	2	10		20	9	1	10	
CA-125 level					0.261					0.296					0.115
≤ 35 U/mL	102	59	14	73	0.113	108	108	8	67	0.283	106	63	6	69	0.063
> 35 U/Ml	36	11	4	15		35	35	4	16		38	11	2	13	
P53					0.198					0.092					0.141
Negative	75	34	13	47	0.890	83	31	8	39	0.108	84	33	5	38	0.082
Positive	63	36	5	41		60	40	4	44		60	41	3	44	
ER					0.631					0.516					0.706
Negative	33	16	6	22	0.853	36	18	1	19	0.630	34	20	1	21	0.736
Positive	105	54	12	66		107	53	11	66		110	54	7	61	
PR					0.604					0.860					0.439
Negative	49	20	6	26	0.353	49	23	3	26	0.651	49	22	4	26	0.722
Positive	89	50	12	62		94	48	9	57		95	52	4	56	
Ki67					0.47					0.117					0.704
Negative	34	14	6	20	0.743	33	20	1	21	0.854	32	20	2	22	0.435
Positive	104	56	12	68		110	51	11	66		112	54	6	60	

*FIGO* International Federation of Gynecology and Obstetrics, *G1* well, *G2* moderate, *G3* poor, *EC* endometrial cancer, *MI* myometrial invasion, *LVSI* lymphovascular space involvement, *LN* lymph node

^a^Two-sided Chi-squared test or Fisher’s test

## Discussion

In this study, we genotyped three polymorphisms in the TNFAIP8 gene, rs11064, rs1045241, and rs1045242, and evaluated their association with its protein expression and with EC risk in women from Heilongjiang Province, China. We found that SNPs rs11064 and rs1045242 in TNFAIP8 gene positively correlated with elevated risk of EC in northern Chinese women. Our studies also indicated significant relationships of SNPs rs11064 and rs1045242 with TNFAIP8 protein expression.

TNFAIP8 locates on chromosome 5q23.1, a 21 kDa cytosolic protein, includes 11 exons and 10 introns, spans about 13.5 KB of genomic DNA. Research has indicated the relationship of TNFAIP8 gene polymorphisms with susceptibility of cervical cancer. TNFAIP8 rs11064 polymorphism especially the variant G allele was associated with cervical cancer risk in Chinese people, indicating a risk allele [8]. Consistently with previous study, our study showed that the G allele of rs11064 increased a 1.791-fold risk for EC in northern Chinese women, which was never reported before. In addition, our study also revealed that the G allele of rs1045242 increased a 1.636-fold risk for EC. Recently, it reported that rs1045241T was related with a high risk of non-Hodgkin’s lymphoma among Chinese population [22].

We also found that the G allele of rs11064 had a possible trend of correlation with advanced FIGO stage, deep myometrial invasion, and lymph node metastasis in EC patients. Our previous study revealed that TNFAIP8 overexpression in ECs correlated with advanced FIGO stage, deep myometrial invasion, lymphovascular space invasion, and lymph node metastasis [10]. These results revealed that SNPs in TNFAIP8 gene might associate with its mRNA and protein expression and increase susceptibility to EC. Although polymorphisms in coding region may change protein expression, numbered researches have explored the association between TNFAIP8 SNPs and its protein expression. Our results suggest that the rs11064 polymorphism positively correlated with TNFAIP8 protein expression, being consistent with that in cervical cancer [8]. However, elaborate mechanisms that intronic polymorphisms affecting protein expression remain unknown.

In addition, our study indicated that the TNFAIP8 rs1045242 polymorphism had a meaningful joint effect with no smoking history and pre-menopausal on strengthening the risk of cancer. Smoking history and the status of menopausal are both important risk factors for EC, but the mechanism underlying this association is unknown. Our results demonstrated that TNFAIP8 may by another way take a part in the development of EC without depending on smoking and status of menopausal. As far as we know, this is the first report investigating the association between TNFAIP8 polymorphisms and the EC risk.

## In conclusion

We firstly evaluate the relationship of TNFAIP8 polymorphisms with its protein expression and with EC risk in women from northern China. This case–control study indicates that SNPs rs11064 and rs1045242 in TNFAIP8 gene are associated with increased risk for EC among northern Chinese women. Furthermore, SNPs rs11064 was associated with TNFAIP8 protein expression. Our findings supply a novel idea, TNFAIP8 disturbing EC, and indicate that TNFAIP8 gene may be an underlying marker for early detection and a target for molecular targeted therapy in EC. Further investigation of these findings is warranted in analyses involving combinations with other alleles.

## Additional files

**Additional file 1: Table S1.** Stratified analysis between TNFAIP8 SNPs and endometrial cancer risk by age.

**Additional file 2: Table S2.** Stratified analysis between TNFAIP8 SNPs and endometrial cancer risk by smoking history.

**Additional file 3: Table S3.** Stratified analysis between TNFAIP8 SNPs and endometrial cancer risk by BMI.

**Additional file 4: Table S4.** Stratified analysis between TNFAIP8 SNPs and endometrial cancer risk by menopausal status.

## Abbreviations

TNFAIP8: tumour necrosis factor-a-induced protein 8
EC: endometrial cancer
SNPs: single nucleotide polymorphisms
OR: odds ratio
HNSCC: human head and neck squamous cell carcinoma
IHC: immunohistochemistry
ER: estrogen receptor
PR: progesterone receptor
CIs: confidence intervals
