# Supplementary material for: Association of TNFAIP8 gene polymorphisms with endometrial cancer in northern Chinese women
Source: Cancer Cell Int. 2019 Apr 23;19:105. doi: 10.1186/s12935-019-0827-9 (PMC6480735; doi:10.1186/s12935-019-0827-9)
Supplement: Supplementary file 1 — Additional file 1: Table S1. Stratified analysis between TNFAIP8 SNPs and endometrial cancer risk by age. [file 12935_2019_827_MOESM1_ESM.docx]

Supplement Table 1. Stratified analysis between TNFAIP8 SNPs and endometrial cancer risk by age

|  | Age (years) | | | | | | |
| --- | --- | --- | --- | --- | --- | --- | --- |
|  | ≤ 54 | | |  | > 54 | | |
|  | case/controls | OR (95%CI) | *^a^P* |  | case/controls | OR (95%CI) | *^a^P* |
| rs11064  AA  AG  GG  AG+GG  rs1045241  CC  CT  TT  CT+TT  rs1045242  AA  AG  GG  AG+GG | 75/93  41/36  9/7  50/43  72/90  43/41  10/5  53/46  75/95  44/40  6/1  50/41 | 1.412 (0.822-2.426)  1.594 (0.567-4.481)  1.442 (0.867-2.398)  1.311 (0.773-2.223)  2.500 (0.818-7.642)  1.440 (0.872-2.379)  1.393 (0.825-2.354)  7.600 (0.895-64.502)  1.545 (0.926-2.578) | 0.211  0.376  0.158  0.315  0.108  0.154  0.215  0.063  0.096 |  | 63/85  29/24  9/3  38/27  71/77  28/31  2/4  30/35  69/82  30/25  2/5  32/30 | 1.630(0.867-3.065)  4.048 (1.053-15.561)  1.899 (1.051-3.430)  0.980 (0.535-1.792)  0.542 (0.096-3.052)  0.930 (0.518-1.668)  1.426 (0.767-2.651)  0.475 (0.089-2.527)  1.268 (0.701-2.292) | 0.129  0.042  0.034  0.947  0.488  0.807  0.262  0.383  0.432 |

^a^Data were calculated by logistic regression, adjusted for age, smoking history, BMI, and menopausal status (excluded the stratified factor in each stratum).

BMI: Body mass index, OR: indicates odds ratio, CI: confidence interval.
