# Supplementary material for: Association of TNFAIP8 gene polymorphisms with endometrial cancer in northern Chinese women
Source: Cancer Cell Int. 2019 Apr 23;19:105. doi: 10.1186/s12935-019-0827-9 (PMC6480735; doi:10.1186/s12935-019-0827-9)
Supplement: Supplementary file 2 — Additional file 2: Table S2. Stratified analysis between TNFAIP8 SNPs and endometrial cancer risk by smoking history. [file 12935_2019_827_MOESM2_ESM.docx]

Supplement Table 2. Stratified analysis between TNFAIP8 SNPs and endometrial cancer risk by smoking history

|  | Smoking history | | | | | | |
| --- | --- | --- | --- | --- | --- | --- | --- |
|  | No | | |  | Yes | | |
|  | case/controls | OR (95%CI) | *^a^P* |  | case/controls | OR (95%CI) | *^a^P* |
| rs11064  AA  AG  GG  AG+GG  rs1045241  CC  CT  TT  CT+TT  rs1045242  AA  AG  GG  AG+GG | 129/166  60/53  17/9  77/62  133/155  61/65  12/8  73/73  134/167  65/56  7/6  72/61 | 1.436 (0.845-2.442)  2.716 (1.010-7.303)  1.620 (0.990-2.649)  1.500 (0.898-2.507)  2.624 (0.859-8.012)  1.614 (0.988-2.635)  1.862 (1.105-3.138)  4.600 (1.224-12.284)  2.032 (1.227-3.367) | 0.181  0.048  0.055  0.122  0.090  0.056  0.020  0.024  0.006 |  | 9/12  10/7  1/1  11/8  10/12  10/7  0/1  10/8  10/10  9/9  1/1  10/10 | 2.245 (0.470-12.526)  0.217 (0.009-5.289)  1.457 (0.272-7.789)  1.344 (0.282-6.398)  -  1.218 (0.260-5.712)  0.917 (0.190-4.430)  0.842 (0.024-29.762)  0.909 (0.197-4.208) | 0.290  0.348  0.660  0.711  1.000  0.802  0.915  0.925  0.903 |

^a^Data were calculated by logistic regression, adjusted for age, smoking history, BMI, and menopausal status (excluded the stratified factor in each stratum).

BMI: Body mass index, OR: indicates odds ratio, CI: confidence interval.
