# Supplementary material for: Association of TNFAIP8 gene polymorphisms with endometrial cancer in northern Chinese women
Source: Cancer Cell Int. 2019 Apr 23;19:105. doi: 10.1186/s12935-019-0827-9 (PMC6480735; doi:10.1186/s12935-019-0827-9)
Supplement: Supplementary file 3 — Additional file 3: Table S3. Stratified analysis between TNFAIP8 SNPs and endometrial cancer risk by BMI. [file 12935_2019_827_MOESM3_ESM.docx]

Supplement Table 3. Stratified analysis between TNFAIP8 SNPs and endometrial cancer risk by BMI

|  | BMI | | | | | | |
| --- | --- | --- | --- | --- | --- | --- | --- |
|  | ≤ 25 | | |  | > 25 | | |
|  | case/controls | OR (95%CI) | *^a^P* |  | case/controls | OR (95%CI) | *^a^P* |
| rs11064  AA  AG  GG  AG+GG  rs1045241  CC  CT  TT  CT+TT  rs1045242  AA  AG  GG  AG+GG | 66/116  25/40  10/7  35/47  63/108  33/50  5/5  38/55  61/115  35/44  5/5  40/49 | 1.125 (0.570-2.219)  2.424 (0.763-7.697)  1.326 (0.714-2.461)  1.419 (0.749-2.689)  3.253 (0.792-13.366)  1.559 (0.844-2.879)  1.646 (0.876-3.093)  2.437 (0.545-10.888)  1.716 (0.934-3.152) | 0.735  0.133  0.372  0.284  0.102  0.156  0.122  0.243  0.082 |  | 72/62  45/20  8/3  53/23  80/59  38/22  7/4  45/26  83/63  39/21  3/1  42/22 | 2.440 (1.123-5.305)  1.944 (0.391-9.668)  2.358 (1.133-4.906)  1.320 (0.622-2.802)  1.356 (0.298-6.177)  1.326 (0.651-2.699)  1.671 (0.772-3.617)  6.699 (0.619-72.482)  1.869 (0.883-3.954) | 0.024  0.417  0.022  0.469  0.694  0.437  0.193  0.118  0.102 |

^a^Data were calculated by logistic regression, adjusted for age, smoking history, BMI, and menopausal status (excluded the stratified factor in each stratum).

BMI: Body mass index, OR: indicates odds ratio, CI: confidence interval.
