# Supplementary material for: Association of TNFAIP8 gene polymorphisms with endometrial cancer in northern Chinese women
Source: Cancer Cell Int. 2019 Apr 23;19:105. doi: 10.1186/s12935-019-0827-9 (PMC6480735; doi:10.1186/s12935-019-0827-9)
Supplement: Supplementary file 4 — Additional file 4: Table S4. Stratified analysis between TNFAIP8 SNPs and endometrial cancer risk by menopausal status. [file 12935_2019_827_MOESM4_ESM.docx]

Supplement Table 4. Stratified analysis between TNFAIP8 SNPs and endometrial cancer risk by menopausal status

|  | Menopausal status | | | | | | |
| --- | --- | --- | --- | --- | --- | --- | --- |
|  | Pre-menopausal | | |  | Post-menopausal | | |
|  | case/controls | OR (95%CI) | *^a^P* |  | case/controls | OR (95%CI) | *^a^P* |
| rs11064  AA  AG  GG  AG+GG  rs1045241  CC  CT  TT  CT+TT  rs1045242  AA  AG  GG  AG+GG | 51/140  28/48  7/7  35/55  49/129  28/59  9/7  37/66  49/142  31/49  6/4  37/53 | 1.601 (0.910-2.819)  2.745 (0.918-8.210)  1.747 (1.027-2.972)  1.249 (0.716-2.181)  3.385 (1.195-9.587)  1.476(0.878-2.282)  1.833 (1.053-3.193)  4.347 (1.177-16.049)  2.023 (1.190-3.440) | 0.103  0.071  0.040  0.434  0.022  0.142  0.032  0.027  0.009 |  | 87/38  42/12  11/3  53/15  94/38  43/13  3/2  46/15  95/35  43/16  2/2  45/18 | 1.586 (0.679-3.706)  1.976 (0.441-8.863)  1.663 (0.763-3.625)  1.626 (0.717-3.685)  0.479 (0.066-3.491)  1.443 (0.661-3.151)  1.187 (0.543-2.597)  0.458 (0.053-3.915)  1.105 (0.517-2.363) | 0.286  0.374  0.201  0.245  0.468  0.358  0.667  0.475  0.797 |

^a^Data were calculated by logistic regression, adjusted for age, smoking history, BMI, and menopausal status (excluded the stratified factor in each stratum).

BMI: Body mass index, OR: indicates odds ratio, CI: confidence interval.
